# Benefits of targeted deployment of physician-led interprofessional pre-hospital teams on the care of critically Ill and injured patients: a systematic review and meta-analysis

**DOI:** 10.1186/s13049-024-01298-8

**Published:** 2025-01-06

**Authors:** Matthew D. Lavery, Arshbir Aulakh, Michael D. Christian

**Affiliations:** 1 Southern Medical Program, Faculty of Medicine, University of British Columbia, 3333 University Way, Kelowna, BC V1V 1V7 Canada; 2 Rural Coordination Centre of BC (RCCbc), 1665 W Broadway Suite 620, Vancouver, BC V6J 1X1 Canada; 3 Department of Critical Care Medicine, Faculty of Medicine, University of British Columbia, Vancouver, Canada

**Keywords:** Pre-hospital care, Out-of-hospital cardiac arrest, Trauma, Resuscitation, Pre-hospital emergency medicine, Pre-hospital physicians

## Abstract

**Introduction:**

Over the past three decades, more advanced pre-hospital systems have increasingly integrated physicians into targeted roles, forming interprofessional teams. These teams focus on providing early senior decision-making and advanced interventions while also ensuring rapid transport to hospitals based on individual patient needs. This paper aims to evaluate the benefits of an inter-professional care model compared to a model where care is delivered solely by paramedics.

**Methodology:**

A meta-analysis and systematic review were conducted using the guidelines of PRISMA 2020. Articles were identified through a systematic search of three databases and snowballing references. A systematic review was conducted of articles that met the inclusion criteria, and a suitable subset was included in a meta-analysis. The survival and mortality outcomes from the studies were then pooled using the statistical software Review Manager (RevMan) Version 8.2.0.

**Results:**

Two thousand two hundred ninety-six articles were found from the online databases and 86 from other sources. However, only 23 articles met the inclusion criteria of our study. A pooled analysis of the outcomes reported in these studies indicated that the mortality risk was significantly reduced in patients who received pre-hospital care from interprofessional teams led by physicians compared with those who received care from paramedics alone (AOR 0.80; 95% CI [0.68, 0.91] *p* = 0.001). The survival rate of critically ill or injured patients who received pre-hospital care from interprofessional teams led by physicians was increased compared to those who received care from paramedics alone (AOR 1.49; 95% CI [1.31, 1.69] *P* < 0.00001).

**Conclusions:**

The results of our analysis indicate that the targeted deployment of interprofessional teams led by physicians in the pre-hospital care of critically ill or injured patients improves patient outcomes.

**Supplementary Information:**

The online version contains supplementary material available at 10.1186/s13049-024-01298-8.

## Introduction

Traditionally, the focus for managing critically ill or injured patients has been on the rapid transport of patients to hospitals to receive ‘definitive care’ delivered by interprofessional teams led by physicians. However, despite this model and significant improvements in in-hospital survival rates for patients, the overall mortality rate due to trauma and critical illnesses, such as cardiac arrest, has changed little as the majority of deaths continue to occur before patients arrive at the hospital [1–3]. Over the past three decades, advanced pre-hospital systems have increasingly integrated physicians to form specialized interprofessional teams. These teams focus on providing early advanced clinical decision-making and clinical interventions beyond the scope of paramedic practice while balancing the need for rapid transport to hospitals based on individual patient needs [4–7]. This paper evaluates the benefits of an inter-professional care model compared to a model where paramedics deliver care alone.

## Methodology

This meta-analysis and systematic review were conducted using the guidelines of PRISMA 2020 (Preferred Reporting Items for Systematic Reviews and Meta-Analyses) [8].

A literature search was conducted for all articles published from 2010 to June 2024 from three databases (PubMed, Medline, and Scopus) based upon predefined search criteria (Supplemental Materials Appendix A). The strategy used the search string: (Pre-hospital OR pre-hospital OR immediate care) AND (Physician OR doctor OR clinician OR "Trauma specialist" OR "general practitioner" OR "Critical care specialist" OR "Emergency medicine specialist") AND ("Critical care" OR trauma OR unstable OR stabilization OR accident OR polytrauma OR stroke OR hemorrhage OR hypothermia OR "cardiac arrest" OR MI) AND (survival OR mortality OR outcome). All reference lists of the included articles were manually reviewed to obtain any relevant articles missed by the initial database search. Experts within the field also identified further articles.

All articles were assessed per the predetermined PICO framework [9] eligibility criteria. If a study met the inclusion criteria below, it was selected and used in the review:

1. Population-The study’s primary population was patients and physicians in pre-hospital care.
2. Intervention: The review included studies that evaluated the impact of the physician-staffed immediate (pre-hospital) care team on patients’ clinical outcomes.
3. Comparator: Care provided by paramedics or other non-physician EMS providers.
4. The primary outcomes of interest included the survival benefit to the patients via improved survival to hospital, survival to discharge, 30 day mortality, or one-year mortality.
5. The review also included only those studies published in English between 2010 and July 2024.

Studies were excluded based on the following exclusion criteria:

1. Studies not published in the English language.
2. Inability to obtain the full text of the article.
3. Studies published before the year 2010.
4. Studies focused primarily on the mode of transport (e.g. ground versus helicopter) as opposed to the model of care delivered.
5. Studies designed as review articles, case reports, and editorials were also excluded from the review.

## Study selection and data extraction

The authors conducted the study selection in different phases. The phases entailed the initial database search, removal of duplicate articles, screening abstracts and titles, and screening of available full texts. An author (ML) first screened the articles' abstracts obtained for inclusion in the review after removing duplicates. If the study met the inclusion criteria, it was included in a shortlist; however, if the reviewer could not ascertain its eligibility, they proceeded to obtain the full text for screening. After completing the shortlisting, two authors (ML, AA) independently reviewed all articles to assess them for inclusion and exclusion criteria and any disagreements were resolved through discussion. The senior author (MDC) reviewed all articles in the shortlist to confirm the appropriate inclusion and exclusion criteria application. One author (AA) then extracted all the relevant data from the included studies. The data extracted from each study included the author ID, the study design, the study setting, the type of intervention, the inclusion criteria, the sample size, the mean age, the male-to-female ratio, the injury severity score (ISS) and the reported outcomes (odds ratios). The summary of the included studies is reported in Table 1.

**Table 1:** Characteristics of the included studies

Author ID	Study setting	Study design	Type of intervention	Study population	Sample size	Age [Mean or median] ± SD (range or IQR)	Male: Female	ISS [Mean or Median] ± SD (range or IQR)	Reported outcomes
Garner et al., 2015^10^	Australia	Randomized controlled trial	Physician-based pre-hospital care	Patients with a GCS score of 3–8 due to blunt trauma as measured in the 1st prehospital tea to arrive at the accident scene	178	43 (26–55)*	124:54	NR	30-day mortality, Persistent vegetative state, or disability
			Standard care		197	40 (27–60)*	153:44	NR	
Hepple et al., 2019^11^	England	Retrospectives analysis	Enhanced critical care team	All injured patients who came to 2 trauma centers. The patients must have either been admitted to critical care, transferred to specialized care within 72 h, or died	281	44.7 (0–97)	206:75	17 (9–29)*	Mortality at hospital discharge
			Non-enhanced care team		281	56.9 (0–105)	195:86	9 (9–19)*	
Lyons et al., 2021^7^	Wales	Retrospective observational study	EMRTS	Patients with traumatic blunt injuries	412	NR	302:110	NR	30-day mortality
			Standard care		3623	NR	1826:1797	NR	
Maddock et al., 2020^12^	Scotland	Retrospective cohort study	Prehospital critical care team (PRCCT)	Adult trauma patients	776	45 (16–95)	565:211	17 (4–75)	30-day mortality
			Non-PRCCT		13,504	56 (16–103)	7641:58:63	9 (1–75)	
Yeguiayan et al., 2011^13^	France	Prospective cohort study	Pre-hospital management by Service Mobile d'Urgences et de Réanimation (SMUR)	Adult patients with severe blunt trauma	2513	41.1 ± 18.0	1910:603	NR	30-day mortality
			Management by non-SMUR (Fire brigade)		193		153:37	NR	
Fukuda et al., 2018^14^	Japan	Retrospective analysis	ALS by a physician	Patients with OHCA	814	58.5 ± 22.2	546:268	NR	1-month survival
			ALS by EMS personnel		814	58.4 ± 22.2	567:247	NR	
Goto et al., 2019^15^	Japan	Prospective observational study	Physician CPR	Patients with out-of-hospital cardiac arrest	16,612	68.2 ± 20.1	10,370:6242	NR	1-month neurologically intact survival
			Paramedic CPR		16,612	68.8 ± 20.1	10,397:6215	NR	
Den Hartog et al., 2015^16^	The Netherlands	Retrospective cohort study	HEMS	Severely injured patients with ISS > 15	681	43 (27–59)*	528:153	26 (22–25)*	Odds of survival at 30-days (adjusted for injury severity)
			EMS		1495	44 (27–62)*	1082:413	22 (17–29)*	
Moors et al., 2019 ^17^	Netherlands	Retrospective observational study	Physician based HEMS	Injured pediatric patients	196	12 (7–16)*	125:71	25 (18–34)*	Odds of survival at 30-days (adjusted for injury severity)
			EMS		112	13 (8–16)*	82:30	19 (17–26)*	
Tsuboi et al., 2024 ^18^	Japan	Retrospective cohort study	Physician staffed GEMS	Patients aged 15–85 years, with an ISS ≥ 16, a clear injury and transport history, and those transported to the hospital from the accident scene	2361	52.7 ± 20.5	1694:667	27.2 ± 10.4	Survival to hospital discharge
			Non-physician staffed GEMS		46,783	56.5 ± 19.7	33,485:13,298	23.5 ± 8.4	
de Jongh et al., 2012 ^19^	Netherlands	Retrospective cohort study	EMS	All patients with an ISS score of between 1 and 75	186	39.9 ± 22.5—with TBI@@36.2 ± 18.2 -without TBI	NR	30.8 ± 11.6—with TBI@@15.5 ± 11.3—without TBI	Mortality at hospital discharge
			EMS/HEMS		186	39.6 ± 22.2 with TBI 36.2 ± 18.8 – with out TBI	NR	33.5 ± 11.0- with TBI 16.0 ± 12.6- without TBI	
Hesselfeldt et l., 2013 ^20^	Denmark	Prospective controlled observational study	Before P-HEMS	Trauma patients with suspected injury. Trauma with reduced level of consciousness.@@Age below 2 years suffering trauma. Mass casualty victims and Horseback riding accidents	56	26 (21–88)^ψ^	39:17	25 (17–45)^ψ^	Odds of survival at 30-days (adjusted for injury severity)
			After P-HEMS		146	47 (15–81)^ψ^	104:44	25 (16–43)^ψ^	
			GEMS		22,203	≥ 15 years	15,694:6504	15–44	
Hagihara et al., 2014 ^21^	Japan	Prospective observational study	Physician staffed ambulances	All OHCA patients without dependent cyanosis, rigor mortis, incineration, or decapitation	9231	69.41 ± 16.93	5894:3337	NR	1-month survival
			Non-physician staffed ambulance		9231	69.44 ± 17.70	5405:3826	NR	
			ACLS by ELSTs		91,559	71.6 ± 17.8	56,507:35,052	NR	
Bujak et al., 2022 ^22^	Poland	Prospective observational study	Physician staffed EMS	All patients aged 18 years and above with OHCA were witnessed by EMS personnel	351	66 (58–77)*	244:107	NR	Survival to hospital discharge
			Paramedic staffed EMS		351	67 (57–77)*	239:112	NR	
Endo et al., 2021 ^23^	Japan	Retrospective cohort study	Physician-led pre-hospital management	Patients aged ≥ 15 years suffering from blunt trauma with an ISS of ≥ 16. Patients are transferred directly from the accident scene. Patients with full data on their transportation to the hospital	2690	62 (41–74)*	1930:760	24 (17–30)	Mortality at hospital discharge
			Paramedic-led pre-hospital management		10,760	62 (43–5)*	7593:3167	24 (17–29)	
Hamilton et al., 2016 ^24^	Denmark	Prospective observational study	Physician-involved prehospital care	All OHCA patients	13,234	70 (59–80)*	8252:4709	NR	1-month survival
			Non-physician-involved pre-hospital care		7931	72 (61–81)*	5052:2879	NR	
Hatakeyama et al., 2023 ^25^	Japan	Retrospective analysis	With a pre-hospital physician	All OHCA patients aged 18 years and above	1173	75.0 (64.0–83.0)*	729:113	NR	1-month survival
			Without a pre-hospital physician		1173	75.0 (63.0–84.0)*	713:460	NR	
Kato et al., 2019 ^26^	Japan	Retrospective cohort study	Physician based EMS	All patients aged ≥ 18 years with OHCA that was not caused by trauma	164	81 (69.8–88)*	82:82	NR	1-month survival
			Paramedic based EMS		718	80 (69–70)*	397:418	NR	
Sato et al., 2019 ^27^	Japan	Retrospective observational study	Physician based care	All adult patients with OHCA of whom CPR had been attempted	135	66 (57–78)*	87:48	NR	1-month survival
			Non-physician-based care		757	78 (66–86)*	519:240	NR	
Obara et al., 2023 ^28^	Japan	Retrospective observational study	Physician present in prehospital care	Patients with OHCA and less than 17 years	276	0–17 years	129:276	NR	1-month survival
			Physician absent in pre-hospital care		911	0–17 years	112:276	NR	
Endo et al., 2020^29^	Japan	Retrospective cohort study	Physician-led pre-hospital management	Patients aged ≥ 15 years suffering from blunt trauma with an ISS of ≥ 16. Patients are transferred directly from the accident scene. Patients with full data on their transportation to the hospital	3032	62 (42–74)*	2156:876	25 (18–32)*	Mortality at hospital discharge
			Paramedic-led pre-hospital management		27,936	63 (43–75)*	19,742:8194	21 (17–27)*	
Hatakeyama et al., 2021^30^	Japan	Retrospective cohort study	Physician present in prehospital care	Patients aged ≥ 18 with OHCA	2186	70 ± 15.3	1438:748	NR	1-month survival
			Physician absent in pre-hospital care		17,061	73.8 ± 14.9	10,513:6548	NR	
Pakkanen et al., 2019^31^	Finland	Retrospective observational study	Physician staffed EMS	Isolated severe TBI presenting with unconsciousness (GCS score ≤ 8)	468	50 (30–64)*	482:169	NR	1-year mortality
			Paramedic staffed EMS		183				

*NR,* not reported, (range), (IQR)*, (5–95% range)^ψ^

## Statistical analysis

The statistical software RevMan Version 8.2.0 was used to perform a meta-analysis. The outcomes analyzed in the analysis included survival and mortality outcomes. Both outcomes were dichotomous; hence, the odd ratio was used in the pooled analysis. Forest plots were then used to present the results. A subgroup analysis according to the patient's category was carried out to determine the benefit accrued by different groups of patients. Our study used a 95% confidence interval (CI) for the meta-analysis, ensuring evaluation of the heterogeneity of the various studies using the I^2^. A low heterogeneity was assigned for I^2^ < 25%, moderate heterogeneity to I^2^ = 25–50%, high heterogeneity to I^2^ > 50%. A random effects model was selected for the meta-analysis, considering the expectations for high heterogeneity of the studies included.

## Quality assessment

The Risk of Bias (ROB)−2 tool was used for the RCTs to analyze the risk of bias across the studies. The ROB-2 assessment tool has five domains, i.e., randomization, deviations, results, and outcome (measurement and reporting). A domain is assigned 'low risk' if the criterion was met correctly, 'some concerns' if the criterion was not addressed correctly, and' High risk' if there was no address to the specified criterion. The overall risk was assigned 'Low' if all the domains had low risk, 'Some concerns' if some domains were assigned some concern, and 'High' if some domains had high risk. On the other hand, the Newcastle Ottawa Scale (NOS) was used in the methodological quality assessment of observational studies. This scale assesses the quality of the studies using three domains: the comparability, selection of participants, and the reporting of the outcomes. The overall quality of the study is then given based on the number of stars the reviewers assign to each domain.

## Results

### Search results

**Fig. 1:**
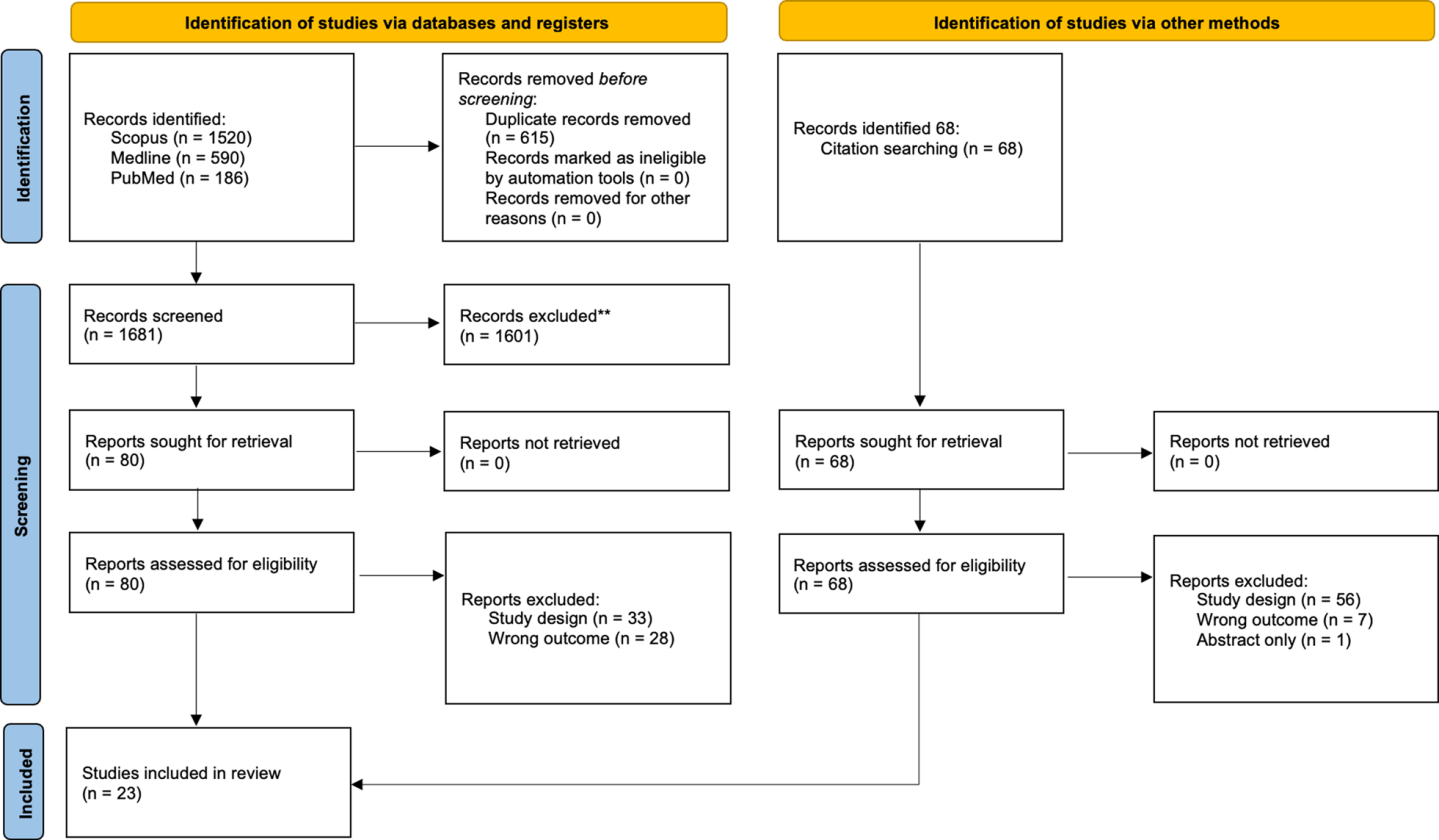
A PRISMA flow diagram summarising the search strategy

Our online search yielded 2296 articles from online databases and 86 from other sources. The initial duplication assessment led to the removal of 615 duplicates. The remaining 1769 publications were assessed based on title and abstract relevance, and 1601 articles were excluded based on their abstract and title irrelevance. One hundred forty-eight articles were sought for retrieval and were retrieved and evaluated using the exclusion and inclusion criteria. After the assessment using our eligibility criteria, we included only 23[7, 10–31] articles in the study and excluded 123 articles that did not meet our inclusion criteria. A PRISMA diagram summarizing the search strategy is outlined in Fig. 1.

## Characteristics of the included studies

This review summarised data from 23 studies, among which 22 were observational and 1 was a RCT. The studies were conducted in various settings, including Japan, the Netherlands, England, Australia, Scotland, Denmark, France, and Wales. The studies included different categories of patients needing pre-hospital care, such as those with traumatic injuries and those with out-of-hospital cardiac arrest. The study's pooled sample size was 332,533 critically ill or injured patients. The characteristics of the included studies are presented in Table 1.

## Characteristics of the comparator care

The care provided in the comparator "non-physician" arms of the studies was generally of a high level of care provided by highly educated clinicians (Supplemental Materials Appendix B). In 22 of the 23 studies, non-physicians provided advanced life-support (ALS) in the comparator arms. Most countries staffed non-physician ambulances with emergency medical technicians (EMT) and ALS providers, most referred to as "Paramedics" or an equivalent translation. The ALS providers had university bachelor's degrees (2–4 years of education) in all countries except Denmark. In Denmark, the minimum training combined five years of pre-hospital clinical experience and two to three years of college education overall. Some countries also offered alternative paths to qualification via vocational training for long-experienced technicians.

## Quality and risk of bias assessment

**Fig. 2:**
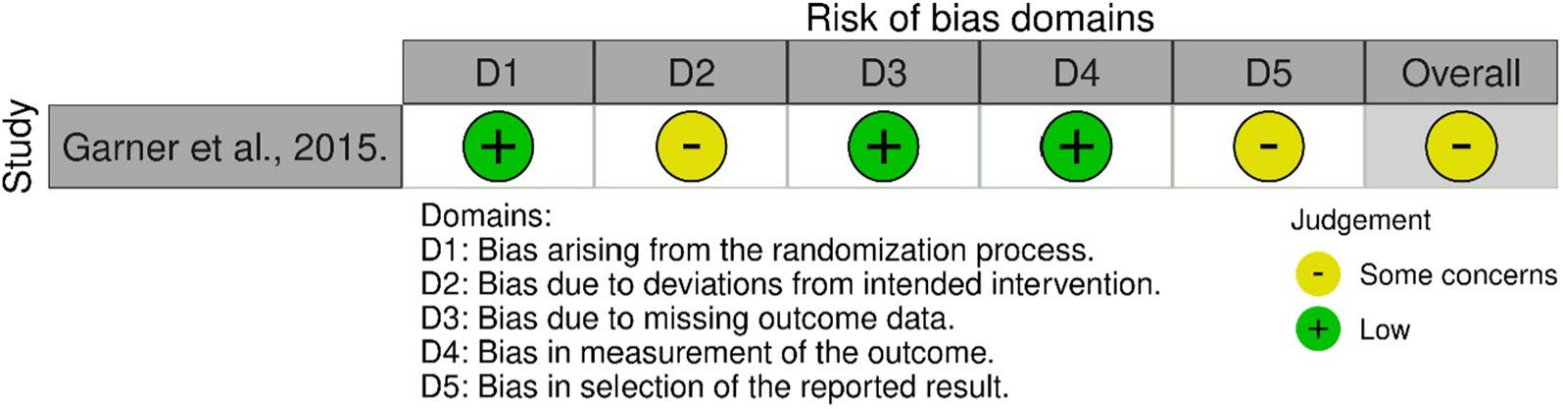
The risk of bias of the included RCT

Due to some concerns under "Bias due to deviations from intended intervention" and "Bias in selection of the reported result," the included RCT had overall "Some concerns" as the risk of biased outcome (Fig. 2).

All the included non-RCT were found to have 'good' methodological quality as evaluated by the Newcastle Ottawa Scale (Table 2).

**Table 2:** The Newcastle Ottawa Scale indicating the methodological quality of the included studies

Author ID	Selection	Comparability	Reported outcomes	AHRQ standard
Hepple et al., 2019^11^	3	2	3	Good
Lyons et al., 2021^7^	3	2	3	Good
Maddock et al., 2020^12^	3	2	2	Good
Yeguiayan et al., 2011^13^	4	2	3	Good
Fukuda et al., 2018^14^	3	2	2	Good
Goto et al., 2019^15^	3	2	3	Good
Den Hartog et al., 2015^16^	3	2	3	Good
Moors et al., 2019^17^	3	2	2	Good
Tsuboi et al., 2022^18^	3	2	3	Good
Hessefeldt et l., 2013^20^	3	2	3	Good
de Jongh et al., 2012^19^	3	2	3	Good
Hagihara et al., 2014^21^	3	2	3	Good
Bujak et al., 2022^22^	3	2	3	Good
Endo et al., 2021^23^	3	2	2	Good
Hamilton et al., 2016^24^	3	2	3	Good
Hatakeyama et al., 2023^25^	3	2	2	Good
Kato et al., 2019^26^	3	2	3	Good
Sato et al., 2019^27^	3	2	2	Good
Obara et al.,2023^28^	3	2	3	Good
Endo et al., 2020^29^	3	2	3	Good
Hatakeyama et al., 2021^30^	3	2	3	Good
Pakkanen et al., 2019^31^	2	2	2	Good

## Mortality outcomes

**Fig. 3:**
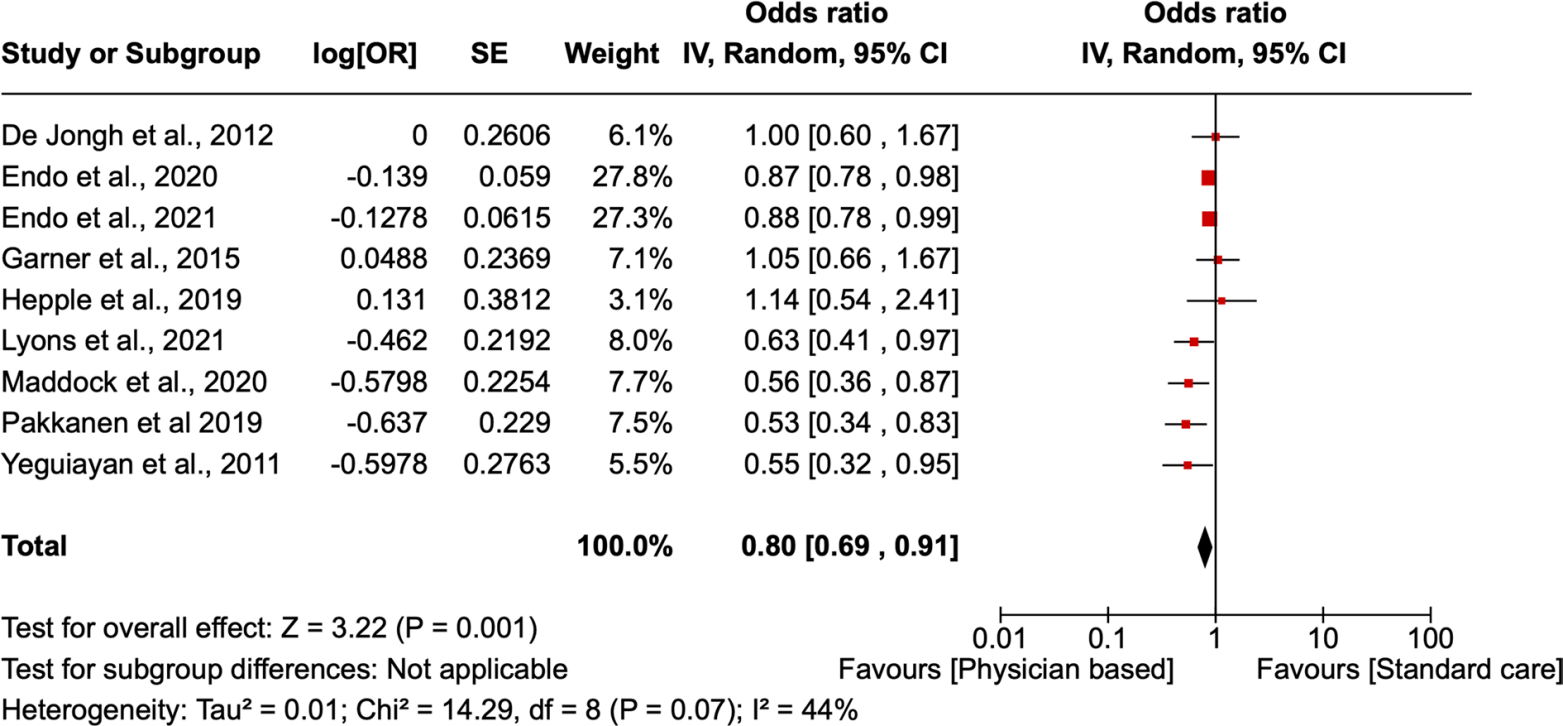
A forest plot showing the mortality outcomes in patients receiving physician-based care compared to standard care

Nine studies reported mortality outcomes in both cohorts of patients. Adjusted odds ratios (AOR) were used to analyze the outcomes, and a pooled analysis of the outcomes showed that physician-led interprofessional team care significantly reduced the mortality of injured patients compared to care from paramedics alone (AOR 0.80; 95% CI [0.68, 0.91] p = 0.001) (Fig. 3).

## Survival outcomes

**Fig. 4:**
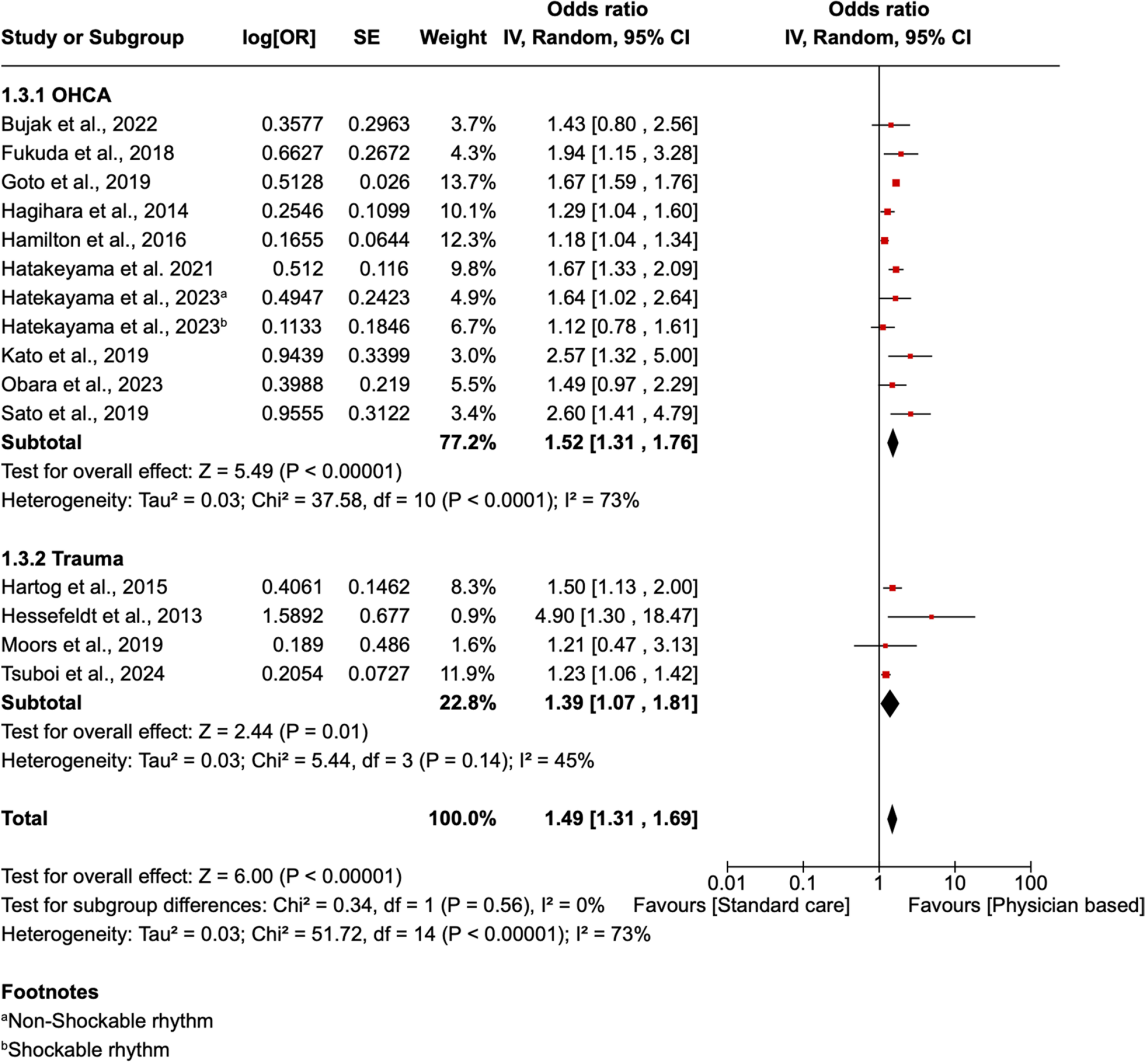
A forest plot showing the survival outcomes in patients receiving physician-based care compared to standard care

Fourteen studies reported survival outcomes. A pooled analysis of the results found that physician-led interprofessional team care increased the survival of critically ill or injured patients compared to care from paramedics alone (AOR 1.49; 95% CI [1.31, 1.69] P < 0.00001). The outcomes had high heterogeneity I^2^ = 73%. A subgroup analysis according to the category of patients indicated that both patients with OHCA and those with major trauma had a significant increase in their survival when they received physician-led interprofessional care compared to when they received care from paramedics alone (AOR 1.52; 95% CI [1.31, 1.76] *P* < 0.00001) and (AOR 1.39 95% CI [1.07, 1.81] *P* = 0.01) respectively (Fig. 4).

## Discussion

Our study analyses outcomes from a pooled sample size of 332,726 critically ill or injured patients who received pre-hospital care and includes 15 new studies published since the most recent meta-analysis, including data up until 2017 [32]. Our study found that interprofessional pre-hospital teams led by physicians significantly decreased in-hospital and 30 days mortality in critically ill or injured patients and increased the survival of major trauma patients. Similarly, a previous review by Knapp et al., 2019 found that the odds of mortality were reduced in severely injured patients who received pre-hospital care from teams including physicians compared to paramedics alone [32]. Furthermore, in the analysis by Knapp et al., when a subgroup analysis was done on the mortality outcomes in studies published after 2005, it was found that the odds were significantly reduced compared to those published before 2005 [32]. Our findings demonstrate that the inclusion of physicians in the provision of pre-hospital care may have evolved over the previous decade with a further reduction in mortality and the additional survival benefits for these patients, which was not addressed in these prior reviews.

In addition to the 2019 systematic review and meta-analysis by Knapp et al., there have been five other large systematic reviews and meta-analyses assessing outcomes of the role of physicians in pre-hospital care, three assessing intubation success rates [33–35] and two assessing outcomes from cardiac arrest [36, 37]. The meta-analyses assessing intubation success rates found that physicians had higher success rates and lower complication rates than paramedics in adult [33, 34] and pediatric [35] patients. Similarly, outcomes were better for pre-hospital patients experiencing either traumatic cardiac arrests [36] or medical cardiac arrest [37] when cared for by interprofessional teams led by physicians. Our subgroup analysis indicated that patients with OHCA had better outcomes when they received pre-hospital care from interprofessional teams led by physicians. This benefit is more prominent in patients presenting in a non-shockable rhythm, as many studies in our review failed to demonstrate an added advantage for patients presenting in a shockable rhythm. The impact of including physicians in interprofessional pre-hospital teams has been assessed in two studies, which found 5.4 additional lives saved per 100 adult patients [38] and 2.5 additional lives per 100 pediatric patients [17].

It is essential to recognize that the care delivered to patients in the studies included in this review involving pre-hospital physicians was not delivered in isolation but in collaboration with paramedics and occasionally nurses in interprofessional teams [39]. In 2010, the World Health Organization highlighted the importance of interprofessional teams in delivering high-quality health care [40]. Numerous studies have documented the benefits of interprofessional team care in critically ill patients in intensive care units, emergency departments, and operating theatres [41, 42]. Interprofessional healthcare teams not only benefit patients but have also been shown to improve the experience for healthcare providers and system-level outcomes for organizations [43]. Despite the well-documented benefits of interprofessional care and the specific evidence reported in our analysis demonstrating the benefit of physician-led interprofessional pre-hospital teams, few emergency medical systems in North America have introduced such teams. Additionally, this stands out as the only phase in the continuum of critical care medicine [44] that rarely incorporates interprofessional practice. Our review’s findings highlight the need to consider further and address potential missed opportunities to improve patient outcomes through the targeted application of physician-led interprofessional teams in these pre-hospital systems.

## Limitations of the current study

The current study aimed to summarise the contemporary literature on the benefits of care delivered by interprofessional pre-hospital teams led by physicians on trauma and other critically ill or injured patients and, therefore, limited the search to articles published after 2010. As a result, the evidence was derived from a subset of the entire body of literature, going back to 1987. Secondly, most of the included articles only analyzed mortality and survival outcomes. However, best practice recommends evaluating interventions against the quintuple aims of healthcare [45, 46]. Currently, insufficient studies on the role of pre-hospital physicians consider outcomes such as provider satisfaction or economic benefits. Finally, because we limited the analysis to manuscripts published in English, there is the potential for selection bias and under-representing global perspectives. However, six of the ten countries represented in the analysis do not have English as their first language, suggesting a good breadth of global perspectives were included in the analysis.

Furthermore, the results were pooled from outcomes of mostly non-randomized studies, with only one RCT included. Thus, the quality of evidence provided by the included studies is of low quality, further limiting the conclusions that can be made from the provided evidence. To generate high-quality evidence, ideally, large prospective RCTs would be undertaken. However, such trials are logistically challenging to conduct. Given the current body of evidence, many clinicians may not feel sufficient clinical equipoise exists to support ethical randomization in a trial. It may, therefore, be only feasible to carry out non-randomized 'natural experiment' studies and retrospective analyses. Consequently, we recommend that future studies optimize their methodological quality and broaden outcomes measured to generate higher-quality evidence.

## Conclusions

Our metanalysis results indicate a significant improvement in the mortality and survival of critically ill or injured patients who receive care from inter-professional teams led by physicians. Furthermore, a subgroup analysis based on the categories of critically ill or injured patients indicated that both patients with OHCA and those with major trauma had survival benefits when a physician was included in their pre-hospital care team. The findings of our review highlight the need to consider the targeted introduction of physician-led interprofessional teams in pre-hospital systems that lack them.

## Supplementary Information

Additional file1.

Additional file2.

Additional file3.

## Data Availability

No datasets were generated or analysed during the current study.
