# Supplementary material for: Benefits of targeted deployment of physician-led interprofessional pre-hospital teams on the care of critically Ill and injured patients: a systematic review and meta-analysis
Source: Scand J Trauma Resusc Emerg Med. 2025 Jan 6;33:1. doi: 10.1186/s13049-024-01298-8 (PMC11702211; doi:10.1186/s13049-024-01298-8)
Supplement: Supplementary file 1 — Additional file1. [file 13049_2024_1298_MOESM1_ESM.docx]

**Appendix A:**

**Question:**

Does implementing a physician-led interprofessional prehospital care team improve survival and reduce mortality for critically ill patients?

**Search terms:**

General:

pre-hospital OR prehospital OR "Immediate Care"

AND

physician OR doctor OR clinician OR "trauma specialist" OR "critical care specialist" OR "emergency medicine specialist"

AND

survival OR mortality OR outcome

AND

"critical care" OR "trauma" OR "unstable" OR "stabilization" OR "accident" OR "polytrauma" OR "stroke" OR "hemorrhage" OR "hypothermia" OR "cardiac arrest" OR "MI"

**Eligibility Criteria:**

- Years (2010-2024)
- Language (English)
- Publication status (Article, Review, meta-analysis, clinical trial)
- Full-text available for review

**Exclusion Criteria**

- Articles which compared only the mode of transport (e.g. helicopters) not the model of care provided
- Physician Staffed ambulance (unless directly comparing to non-physician staffed)

**Information sources :**

Databases (PubMed, Medline, and Scopus)
