# Supplementary material for: Benefits of targeted deployment of physician-led interprofessional pre-hospital teams on the care of critically Ill and injured patients: a systematic review and meta-analysis
Source: Scand J Trauma Resusc Emerg Med. 2025 Jan 6;33:1. doi: 10.1186/s13049-024-01298-8 (PMC11702211; doi:10.1186/s13049-024-01298-8)
Supplement: Supplementary file 2 — Additional file2. [file 13049_2024_1298_MOESM2_ESM.docx]

Appendix B: Characteristics Comparator Care.

| **Author ID.** | **Study setting** | **Description of comparator care** | **Clinicians*** | **Education*** | **Scope(s) of Practice*** |
| --- | --- | --- | --- | --- | --- |
| Garner et al., 2015 ^10^. | Australia | Standard care by paramedics only was according to written proto-  cols of the New South Wales Ambulance including: cannulation and up to 1-litre intravenous crystalloid infusion; ventilation via supraglottic airways and bag-valve-mask ventilation; intubation  without neuromuscular blockade; needle chest decompression; midazolam for seizures or sedation; analgesia with methoxyflurane and morphine and splinting and spinal immobilisation.  Monitoring consisted of pulse oximetry, ECG and manual BPs. | Paramedics | **Paramedic** = 3-4 yr Bachelor’s degree | ALS |
| Hepple et al., 2019^11^. | England | Details not provided beyond the standard ambulance response in Northeast England by non-enhanced prehospital care teams. | EMTs,  Paramedics | **EMT** = 12-18 months vocational training  **Paramedic** = 2-4-year Bachelor’s degree | ALS |
| Lyons et al., 2021^7^. | Wales | Details the additional skills provided by the intervention rather than describing the comparator care in detail stating “EMRTS provides critical care interventions outside standard ambulance practice such as the administration of blood products and anaesthesia at the scene for both illness and injury (full details available in online Supporting Information Appendix [S1](https://associationofanaesthetists-publications.onlinelibrary.wiley.com/doi/10.1111/anae.15457#support-information-section)).” | EMTs,  Paramedics,  SPs  APPs | **EMT** = 12-18 months vocational training  **Paramedic** = 2-4-year Bachelor’s degree  **Specialist Paramedic** = 6-12 month post-graduate diploma  **APP** = 1-2 year Master’s of Science degree | BLS  ALS |
| Maddock et al., 2020^12^. | Scotland | “The vast majority  of paramedics in Scotland do not have enhanced critical care skills.”  “In more rural areas, the Scottish Ambulance Service’s response  may be augmented by voluntary responder schemes such as those of BASICS (the British Association for Immediate Care) Scotland, meaning that some patients will be seen by a non-critical care doctor (often a local General Practitioner) or nurse in addition to standard (non-PHCCT) ambulance care.” | EMTs,  Paramedics  +/- RNs  +/- GPs | **EMT** = 12-18 months vocational training  **Paramedic** = 2-4-year Bachelor’s degree | BLS  ALS  +/- CC |
| Yeguiayan et al., 2011^13^. | France | “The fire brigade will provide only basic life support and take the patient to the closest hospital.” | Fire Fighters | N/A | BLS |
| Fukuda et al., 2018^14^. | Japan | “ Most ambulances include at least 1 emergency life-saving technician (ELST), certified to insert an intravenous catheter and a supra-glottic airway device (since 1991). In addition, a specially trained ELST, who has completed an extensive training program, can administer epinephrine and insert an endotracheal tube. Epinephrine administration and endotracheal intubation by specially trained ELST officially started in April 2006 and July 2004, respectively. As of 2014, almost all (97.4%) ambulances included at least 1 ELST, and most (82.9%) were specially trained ELSTs.” | BEMTs  ELSTs | **BEMT** = 250h vocational training  **ELST** = 2000h experience as BEMT + 6 months training or 2-4 years Bachelor’s degree | BLS  ALS |
| Goto et al., 2019^15^. | Japan | “Most local governments use a one-tiered system, with a crew of three EMS personnel including at least one emergency lifesaving technician. Emergency lifesaving technicians are permitted to use several resuscitative methods, including automated external  defibrillators, insertion of an airway adjunct or a peripheral intravenous  line, and administration of Ringers lactate solution. However, only specifically emergency lifesaving technicians are permitted to insert a tracheal tube and administer intravenous adrenaline (epinephrine)” | BEMTs  ELSTs | **BEMT** = 250h vocational training  **ELST** = 2000h experience as BEMT + 6 months training or a 2–4-year Bachelor’s degree | BLS  ALS |
| Den Hartog et al., 2015^16^. | Netherlands | “EMS units are staffed with paramedics, all of which are trained in prehospital  trauma life support (PHTLS). EMS paramedics are not allowed to  perform all Advanced Trauma Life Support (ATLS) procedures; for instance, they are not allowed to use general anaesthesia for  intubation or to insert a chest tube.” | EMTs  Paramedics | **EMT** = 12-18 months vocational training  **Paramedic** = 4-year Bachelor’s degree + 9 month post-grad certificate. | ALS |
| Moors et al., 2019 ^17^. | Netherlands | “ Prehospital medical care is primarily provided by an emergency medical service (EMS) system covering the entire country and staffed by paramedics. These paramedics are registered nurses, of whom the vast majority have a background of intensive care unit, emergency department, or anesthesia departments.  They receive an additional training of 9 months.” | EMTs  Paramedics | **EMT** = 12-18 months vocational training  **Paramedic** = 4-year Bachelor’s degree + 9 m post-grad certificate. | ALS |
| Tsuboi et al., 2024 ^18^. | Japan | “Through non-physician stafed ground emergency medical services (GEMS) in Japan, paramedics provide pre-hospital trauma care via oxygen administration, spinal immobilization, compression hemostasis, and cardiopulmonary resuscitation. Under a doctor's guidance, paramedics are now able to provide advanced frst-aid measures for cardiopulmonary arrest, including semi-automatic defbrillation, tracheal intubation, pre-hospital lactated Ringer's solution treatment, and adrenaline administration” | BEMTs  ELSTs | **BEMT** = 250h vocational training  **ELST** = 2000h experience as BEMT + 6 months training or a 2–4-year Bachelor’s degree | BLS  ALS |
| De Jongh et al., 2012 ^19^. | Netherlands | “In the Netherlands, all emergency medical services (EMS) are staffed by paramedics who have years of clinical experience and education (e.g., in intensive care or emergency medicine), supplemented with three additional years of specific ambulance training. All paramedics are Pre-Hospital Trauma Life Support (PHTLS) certified.” | EMTs  Paramedics | **EMT** = 12-18 months vocational training  **Paramedic** = 4-year Bachelor’s degree + 9 month post-grad certificate. | ALS |
| Hesselfeldt et l., 2013 ^20^. | Denmark | “The existing regional EMS system consisted of ground units staffed with personnel on three competence levels. Level 1 is basic life support providers. Level 2 and 3 providers are all pre-hospital  trauma life support certified with differentiated authority to administer intravenous fluid and medication. None has competence in tracheal intubation.  Level 3 (paramedics) providers are allowed to insert laryngeal mask airway.” | Ambulance Assistants  Technicians (EMT)  Paramedics | **Ambulance Assistant** = 12 months college education  **Technician (EMT)** = minimum 1.5 year’s experience experience as an ambulance assistant then completing 6-12 months college education  **Paramedics** = minimum 5 years ambulance experience with a minimum of 3 years as an EMT, then completing 6 months college education | ALS |
| Hagihara et al., 2014 ^21^. | Japan | “An ambulance crew consisted of three emergency providers, including at least one emergency life-saving technician”  “The certifying paramedic [BEMT *sic*] curriculum in Japan generally includes 180 h of lectures and practice in school and experience in 30 successful cases in the operating room under the instruction of an anesthesiologist.”  “Emergency life-saving technicians are  permitted to insert adjunct airways and to use semi-automated external defibrillators.”  “Specially trained emergency life-saving  technicians have been permitted to insert intravenous lines since July 2004, and certified emergency life-saving technicians have been permitted to administer intravenous epinephrine since April 2006”. | BEMTs  ELSTs | **BEMT** = 250h vocational training  **ELST** = 2000h experience as BEMT + 6 months training or a 2–4-year Bachelor’s degree | BLS  ALS |
| Bujak et al., 2022 ^22^. | Poland | “In the case of OHCA, paramedics and EMS nurses in Po-  land are credentialed to perform procedures recommend-  ed by the European Resuscitation Council (ERC) guidelines on advanced life support (ALS), i.e., using a manual defi-  brillator, securing the airway with either a tracheal tube or supraglottic devices, and administering resuscitation drugs. Moreover, according to Polish law, paramedics and EMS nurses are allowed to withhold or terminate CPR.  Furthermore, in Poland, the first-responder system has not been widely implemented, except for the  firefighters of the State Fire Service, who are trained for CPR, including the use of an automated external defibrillator  (AED), and may be dispatched by the dispatcher to initiate CPR when a long delay to EMS arrival is expected.” | Paramedics  RNs | **Paramedic** = 3-year Bachelor's degree  **RN** = 3-year (6 semester) Bachelor's degree | BLS  ALS |
| Endo et al., 2021 ^23^. | Japan | “ Japanese paramedics responding to trauma patients without cardiac arrest are limited to performing spinal motion restriction, external fixation  of bone fractures, oxygen administration using a mask, and administration of Ringer’s solution (only to patients  with shock).” | BEMTs  ELSTs | **BEMT** = 250h vocational training  **ELST** = 2000h experience as BEMT + 6 months training or a 2–4-year Bachelor’s degree | BLS  ALS |
| Hamilton et al., 2016 ^24^. | Denmark | “Emergency ambulances equipped with defibrillators and staffed with either emergency medical technicians (EMTs) who can perform basic life support or paramedics who can give advanced life support.”  “ Paramedics and other EMS personnel operate under standing orders and protocols.” | Assistants  Technicians (EMT)  Paramedics | **Ambulance Assistant** = 12 months college education  **Technician (EMT)** = minimum 1.5 year’s experience experience as an ambulance assistant then completing 6-12 months college education  **Paramedics** = minimum 5 years ambulance experience with a minimum of 3 years as an EMT, then completing 6 months college education | BLS  ALS |
| Hatakeyama et al., 2023 ^25^. | Japan | “Each ambulance has three personnel, at least one of whom is an emergency life-saving technician who has undertaken advanced training in the provision of pre-  hospital emergency care. All emergency life-saving technicians are allowed to insert an intravenous line and an adjunct airway. The specially trained emergency life-saving technicians are permitted to insert tracheal tubes for patients with cardiac arrest. However, they are not able to insert tracheal tubes after the return of spontaneous circulation. They also are permitted to inject intravenous adrenaline.” | BEMTs  ELSTs | **BEMT** = 250h vocational training  **ELST** = 2000h experience as BEMT + 6 months training or a 2–4-year Bachelor’s degree | BLS  ALS |
| Kato et al., 2019 ^26^. | Japan | “Three EMS staff members are assigned to  an ambulance, and all EMS personnel are able to provide cardiopulmonary resuscitation (CPR) for basic life support  and have been trained to use semiautomatic external defibrillators and airway devices. At least one emergency life-saving technician is assigned to each ambulance. Technicians are allowed to provide extensive resuscitation care, for example, they can give adrenaline through venous infusion lines and use advanced airway management, including intra-  tracheal intubation” | BEMTs  ELSTs | **BEMT** = 250h vocational training  **ELST** = 2000h experience as BEMT + 6 months training or a 2–4-year Bachelor’s degree | BLS  ALS |
| Sato et al., 2019 ^27^. | Japan | “A standard ambulance is staffed with three crewmembers, including at least one ELST. These crewmembers are trained in rescue, stabilisation, transportation and advanced care in traumatic and medical emergencies”  “Two of the three crewmembers  are qualified ELSTs who are permitted to use invasive alternative airways, such as a laryngeal mask airway and the Combitube, to treat patients with cardiac arrest.  For those who have completed the Additional National Standard Training course, tracheal intubation is the  treatment of choice in the management of patients with cardiac arrest. Authorised ELSTs are able to administer epinephrine.” | BEMTs  ELSTs | **BEMT** = 250h vocational training  **ELST** = 2000h experience as BEMT + 6 months training or a 2–4-year Bachelor’s degree | BLS  ALS |
| Obara et al., 2023 ^28^. | Japan | “Each emergency ambulance is staffed by three or four EMS crew members, at least one of whom is a highly trained staff member known as an ELST. ELSTs were authorized to provide advanced life support, including placement of supraglottic airway devices. Specially trained ELSTs were allowed to perform tracheal intubation and administer IV adrenaline.”  “Intraosseous access by EMS personnel was not permitted.” | BEMTs  ELSTs | **BEMT** = 250h vocational training  **ELST** = 2000h experience as BEMT + 6 months training or a 2–4-year Bachelor’s degree | BLS  ALS |
| Endo et al., 2020^29^. | Japan | “Medical interventions allowed to Japanese paramedics responding to trauma patients without cardiac arrest are limited to performing spinal motion restriction, external fixation  of bone fractures, oxygen administration using a mask, and administration of Ringer’s solution (only to patients  with shock).” | BEMTs  ELSTs | **BEMT** = 250h vocational training  **ELST** = 2000h experience as BEMT + 6 months training or a 2–4-year Bachelor’s degree | BLS  ALS |
| Hatakeyama et al., 2021^30^. | Japan | Referenced previous publications included above. | BEMTs  ELSTs | **BEMT** = 250h vocational training  **ELST** = 2000h experience as BEMT + 6 months training or a 2-4-year Bachelor’s degree | BLS  ALS |
| Pakkanen et al., 2019^31^. | Finland | “Paramedic-staffed EMS units provided pre-hospital care in this region. During the study period, patients with a decreased level of consciousness were routinely administered oxygen according to national guidelines. Neuromuscular blocking agents were not available and pre-hospital advanced airway management was per-  formed using sedatives and opioids only.” | Paramedics | **Paramedic** = 4-year Bachelor’s degree | ALS |

* Clinical staffing, education and scope of practice reported as at the time of the study based upon published reports, websites, manuscripts and direct correspondence with EMS leaders within the countries.

**Abbreviations**

APP = Advanced Paramedic Practitioner

ALS = Advanced Life Support

BEMT = Basic Emergency Medical Technicians

BLS = Basic Life Support

CC = Critical Care

EMT = Emergency Medical Technician

ELST = Emergency Life-Saving Technician

GP = General Practitioner (Physician)

N/A = Not available

RN = Registered Nurse

SFAC = Standard First Aid Class

SP = Specialist Paramedic

**Definitions**

Basic Life Support (BLS) includes skills or procedures such as automated external defibrillation, oxygen administration, CPR, and supraglottic airways.

Advanced Life Support (ALS) includes skills or procedures such as administering parenteral (IV/IO) fluids and medications, defibrillation, and endotracheal intubation.

Critical Care (CC) includes skills or procedures such as prehospital emergency anaesthesia, surgical procedures, and blood product administration.
